# Exploring the usefulness of indicators for referring people with dementia and their informal caregivers to activating interventions: a qualitative analysis of needs assessments

**DOI:** 10.1186/s12877-019-1221-0

**Published:** 2019-08-23

**Authors:** Netta Van’t Leven, Jacomine de Lange, Johanna Groenewoud, Eva van der Ploeg, Anne Margriet Pot

**Affiliations:** 1 Research Centre Innovations in Care, Rotterdam University of Applied Sciences, Rochussenstraat 198, 3015 EK Rotterdam, The Netherlands; 2 Department of Public Health & Primary Care, Leiden University Medical Center, Leiden, The Netherlands; 3 Department of Clinical Psychology, Vrije Universiteit Amsterdam, Amsterdam, The Netherlands; 4 Amsterdam Public Health research institute, Amsterdam, The Netherlands

**Keywords:** Dementia, Caregivers, Activity needs, Person-centred care, Psychosocial interventions, Exercise, Occupational therapy, Pleasant events

## Abstract

**Background:**

People with dementia (PWDs) and their informal caregivers frequently report difficulties in maintaining their usual activities. We had previously developed a set of indicators to estimate whether dyadic, activating interventions can meet these needs for activity. This study investigates how PWDs and informal caregivers talk about the indicators in interviews for needs assessments, and how professionals identify activity needs and preferences. Our research goal was to explore the usefulness of the indicators for assessing the activity needs of community-dwelling dyads. Such assessments are needed for appropriate referral to activating interventions.

**Methods:**

A dementia case manager assessed the needs of community-dwelling PWDs and their informal caregivers; we carried out secondary analyses on the dataset resulting from the audio-tapes and transcripts. We applied qualitative, deductive content analysis because we wanted to identify both explicit and implicit needs and preferences. We used the indicators that we had developed in previous research as codes.

**Results:**

Both PWDs and informal caregivers do explicitly mention needs, preferences, and characteristics related to the indicators in the needs assessments. Possible implicit needs and preferences were frequently identified in their stories.

**Conclusions:**

Needs-driven care requires high-quality needs assessments. Both PWDs and their informal caregivers need encouragement to express their latent needs and preferences. In addition, latent needs and preferences have to be further explored in needs assessments to find out the real meaning. The outcomes of this study highlight the significance of structured needs assessments for mapping the activity needs of PWDs and their informal caregivers. Many PWDs and informal caregivers reported activity needs, which suggests that activating interventions may be appropriate. The indicators can help professionals identify activity needs so that they can discuss matching activating interventions with the dyad.

**Electronic supplementary material:**

The online version of this article (10.1186/s12877-019-1221-0) contains supplementary material, which is available to authorized users.

## Background

People with dementia (PWDs) encounter all kinds of difficulties in maintaining their activities of daily living and social functioning. They need support and care to manage their everyday life [1]. Informal caregivers often provide most of the care and support for community-dwelling PWDs [2]. They may have difficulties helping the PWDs with daily activities and maintaining their own activities at the same time due to stress and a lack of time [2, 3]. The PWDs and their informal caregivers often have difficulty adapting the daily routine to their changing capacities [4–6].

Dyadic activating interventions aim to increase a dyad’s skills to continue meaningful activities and cope with diminishing capacities [7–11]. The overall evidence for their efficacy is heterogeneous [12, 13]. Nevertheless, dyads who participated in these interventions, felt empowered by having been offered solutions for their most important activity needs. Such solutions came from in-depth assessments of their capacities and limitations [14]. After disclosure of the diagnosis, PWDs receive little advice about dyadic activating interventions that focus on maintaining daily functioning and social roles [15, 16].

Dyadic activating interventions should match dyads’ needs, characteristics, and preferences [17, 18]. Individual needs and preferences for support vary largely, depending on the personal setting, health and comorbidity, and the coping style of both the PWD and their informal caregiver [19–21]. In a qualitative study, we found that dyads who wish to remain active and are open to alternative ways of going about their daily activities, might profit from these interventions [22]. Identifying activity needs, personal characteristics, and preferences is a prerequisite for estimating the appropriateness of interventions for a dyad [17]. Because needs and preferences may be latent or expressed implicitly, probing questions can help reveal them [23, 24].

To help professionals explore the activity needs, characteristics, and preferences of dyads, we developed a set of indicators for referral on the basis of the findings of the qualitative study. The set of 31 indicators was divided into five themes: ‘Need for activities’, ‘Timing and openness’, ‘Lifestyle’, ‘Doing things apart or together’ and ‘Meaning of activities’ (Table 1).

**Table 1:** Presence of indicators in needs-assessment

Indicators		Present
1. Need for activities		
1	The PWD has a need for a meaningful occupational routine	14
2	The CG has a need for advice how to cope with the behavior of the PWD	9
3	The CG has a need for support in how to assist or instruct the PWD to perform activities	6
4	The CG has a need for more insight into the capacities of the PWD, what he or she is able to do	1
2. Timing and openness for change.		
1	The dyad is informed sufficiently about the consequences of dementia for daily life	4
2	The CG has an understanding of the consequences of dementia for the daily activities of the PWD	2
3	The dyad has a pro-active attitude, they want to anticipate themselves on future situations	7
4	The dyad wants to counteract decline actively as much as possible	–
5	The dyad is not focused on limitations, but on possibilities	9
6	The dyad wants to strongly maintain their current way of living	3
7	The PWD still has the capacity to cut out with routines (e.g. use a memory-aid)	–
8	The CG is able to put energy into coping with a new approach, is not overburdened	1
3. Lifestyle		
1	The dyad or one of them (PWD or CG) has or had an active lifestyle	8
2	The PWD and/or CG likes physical activity	15
3	The PWD and/or CG likes doing sports, in an institutional (group) setting or as a routine (running, bicycling)	6
4	The PWD and/or CG is used to sport	5
5	The PWD and/or CG likes outings like shopping or making a visit	9
4. Apart or together		
1	The PWD is/is not accustomed to spend time alone	–
2	The PWD depends a lot/ limited on CG during the day	7
3	The PWD likes to have enjoyable shared activities with CG	3
4	The CG has a need for enjoyable shared activities with PWD	6
5	The CG has a strong/limited need for his or her own activities	8
6	The CG has a need for more time for his or her own life	10
5. Meaning of activities		
1	The PWD has a strong/limited need for something to do for passing time, See 1.1.	14
2	The PWD has a strong/limited need for physical activity, See 3.2.	15
3	The PWD has a strong/limited need for social contacts	5
4	The PWD has a strong/limited need for self-sufficiency	7
5	The PWD has a strong/limited need for positive experiences	–
6	The PWD will benefit from adaptations or assistive devices for physical limitations	8
7	The CG has a need for advice about safety at home	2
8	The CG has a need for advice about safety outside	2

*PWD* person with dementia, *CG* caregiver

The theme ‘Need for activities’ appeared to be the central theme, and the other themes provide guidance for determining the type of activities that may be most appropriate. The indicators of ‘Timing and Openness’ are related to acceptance and readiness for interventions that make use of adaptations of activities and environment, and encourage changing daily routines. The ‘Lifestyle’ indicators facilitate choosing the type of activities that motivate the dyad. The indicators of ‘Doing things apart or together’ relate to preferences and routines of a dyad in their relationship and to the changing interdependence between the PWD and informal caregiver. The indicators concerning the ‘Meaning of activities’ may help in identifying the goals and objectives that are important to the dyad, such as having a fixed routine in the day, just passing time doing something at hand, being physically active, maintaining social contacts, maintaining independency, addressing safety inside and outside the house, or addressing physical limitations that hinder activities. A panel of clinicians recognised most of the indicators in their clinical practices [25]. The panel confirmed that needs assessment is a multi-layered process in which needs and preferences often have to be coaxed out of the dyad’s stories [25]. To explore the usability of the indicators in clinical practice, the current study investigated how PWDs and their informal caregivers talked about the indicators in needs assessments and how professionals identified these needs and preferences. Our research goal was to explore the usefulness of indicators in assessing the activity needs of community-dwelling people with dementia and their informal caregivers for appropriate referral to activating interventions.

## Methods

### Study design

We used a qualitative approach to identify needs, preferences, and characteristics as described in the indicators [26]. Latent needs and preferences can be interpreted from the stories told in the interviews [27]. For this study, we used data collected for the VitaDem project in our secondary analysis. The integral needs-driven approach developed in the 3-year VitaDem project (2015–2018) was intended to enhance functional independence and social inclusion of community-dwelling PWDs and their informal caregivers [28–30]. The purpose of the needs assessment in the VitaDem project was to reveal individual needs and the dyad’s shared needs of self-sufficiency, vitality, and social inclusion. Needs to maintain daily activities were part of the semi-structured needs-assessment interviews, by dementia case managers. The level of education of the dementia case managers was comparable to higher professional education, completed with clinical experience (1–25 years). For this project they were trained to explore the stories of the PWDs and caregivers in depth and to avoid giving solutions before exploration. The PWDs and caregivers were interviewed separately in their homes. A multi-disciplinary case conference followed for discussion of the principal needs and wishes of the dyad and brainstorming about the most appropriate tailored interventions, after clarification of these needs and wishes.

### Ethical considerations

The Medical Ethics Review Committee of the Erasmus Medical Center, the Netherlands, gave their ethical approval for the VitaDem project (MEC-2015-028). The PWDs and informal caregivers consented to audio recording of the interviews and anonymous use of the data for research.

### Data and study population

Our analysis included transcripts of the two interviews for each dyad and the first part of the case conference, in which the case manager answered questions to clarify dyads’ needs. Complete data with the needs assessments of 20 dyads were available.

The case managers recruited dyads from their client databases. The inclusion criteria were: the PWD had to have a diagnosis of dementia, both the PWD and their informal caregiver had to be 65 years old or older, and both had to be living in the case manager’s practice area. The exclusion criteria were inability of the candidates to express themselves in Dutch or being on a waiting list for institutionalisation (Table 2).

**Table 2:** Study population

	Persons with dementia	Caregivers
Mean age (range)	81 years (68–89)	76 years (48–84)
Men/Women	14 Men/6Women	5 Men/15Women
MMSE Mean (range)	21.5 (10–29)	
Dyad- relation	19 spouses 1 mother-daughter	

*MMSE* Mini Mental State Examination

There was an even distribution in education level, employment history and income in the study population. The needs-assessment interviews with PWDs lasted averagely 34 (12–80) minutes. The interviews with their informal caregivers lasted averagely 41 (18–75) minutes. The clarification part of the case conferences lasted averagely 15 (10–25) [10–25] minutes. The interviews and case conferences took place between April 2015 and March 2017. The interviews and multi-disciplinary case conferences were transcribed verbally, anonymised, and imported into Atlas-ti 7.2 for qualitative analysis.

### Data analysis

We used deductive content analysis to explore how the indicators appeared in the needs assessments [31]. We carefully read three transcripts per dyad (two interviews and the clarification in the case conference), and we listened to the audio recordings of the interviews to familiarise ourselves with the stories. We sought text fragments that represented a need, characteristic, or preference related to the indicators and used the indicators as codes. The text fragment could present an explicitly mentioned present or absent need, characteristic, or preference, and it could refer to a latent need or implicit preference. Three researchers (NL, JL, and a trainee) independently coded the transcripts for the first dyad, and they discussed differences in linking text fragments to indicators. Then NL and the trainee coded the transcripts for 10 dyads. NL coded the remaining transcripts. We summarised the text fragments for indicators into a condensed description, illustrated with quotes. After analysing the needs assessments for 16 dyads, we discussed the preliminary results for their plausibility and consistency with the researchers involved in the VitaDem project (NL, JL, JG, and a researcher). To make the trustworthiness more rigorous, three researchers (JL, JG, and a researcher) coded the transcripts for one dyad independently to check the consistency with NL’s coding. The three researchers coded nine of the ten indicators that NL had coded in that the same transcript, and we concluded that our coding was consistent to a great extent. We counted the presence of the indicators as the last step.

## Results

PWDs and their informal caregivers do mention needs, preferences, and characteristics related to the indicators in the needs-assessment interviews (Table 1). Most presentations of the indicators related to the (former) ‘Lifestyle’ were explicit and clear. The indicators related to the other themes, however, were more often implicitly present in the clients’ stories. These needs and preferences should have had further exploration in the interviews to find out the real meaning. In total, we identified 27 of the 31 indicators.

### The ‘Need for activities’ theme

The indicator describing the PWD’s need to maintain activities was present in the needs assessments of 14 dyads. Some PWDs said quite clearly that they wanted to keep doing what they were used to doing, and some mentioned specific activities, such as household activities or a hobby. A latent need for activity was recognised when a PWD said that he or she was bored or missed a daily structure. Other PWDs said that everything was going fine; they did not know of anything more they could wish for. However, their excitement when talking about past activities might suggest a latent need for current activities. Caregivers had a need for advice about how to help the PWDs. Some were irritated by the PWD’s behaviour; for example, about the PWD’s inactivity or a PWD gardening for hours without a pause for a meal. These examples may indicate a need for help in learning how to assist the PWD in activities, although the case managers did not explore this topic further.

### The ‘Timing and openness’ theme

Several dyads said that they gathered information about dementia, some has already adapted their daily routines, and some said that they took future changes into account. Rejecting domestic help because one prefers to do it one’s own way can represent a strong wish to stick to one’s current ways.

### The ‘lifestyle’ theme

The PWDs and informal caregivers clearly detailed their (former) lifestyles: how they spent their days, what their usual activities were, and which outings or sports they preferred.

### The ‘Doing things apart or together’ theme

Many informal caregivers clearly stated their need to maintain their own activities. Several dyads also mentioned the need for enjoyable shared activities. Some informal caregivers said they felt a lack of time for shared activities because the PWD attended day-care and had other appointments with care services.

### The ‘Meaning of activities’ theme

Several PWDs and informal caregivers explained clearly what the activities meant to them, and why the activities were important to them. However, the nature of the activities (e.g., social contact, being self-sufficient, or safety) was frequently unclear and difficult to identify from the stories in the needs assessments. One PWD said that he did not miss the gardening after he gave up his allotment garden, but when encouraged to tell more, he said he missed the mate he collaborated with. Another PWD said he wanted to continue driving; probing questions made it clear that driving a car meant for him that he could keep his role of helping his wife get the groceries.

Some indicators overlapped or were difficult to distinguish from each other. The indicators about the caregiver’s need for advice about how to cope with behaviour (Table 1-1.2) and how to assist the PWD in activities overlapped (Table 1-1.3). The indicators concerning the meaning of activities for passing time (Table 1–5.1) and engaging physical activity (Table 1–5.2) were difficult to distinguish from indicators belonging to the themes of ‘Need for activities’ (Table 1-1.1) and ‘Active lifestyle’ (Table 1–3.2).

See ‘*Availability of data and materials*’ for an Additional file 1 with a complete overview of the representations of all indicators in the needs assessments.

## Discussion

This study shed light on how community–dwelling PWDs and their informal caregivers expressed their needs and preferences for maintaining meaningful activities, and how case managers identified these needs and preferences. Our study confirms that dyads often have needs to maintain activities. However, they need encouragement to express and to explore those needs. We frequently identified activity needs and preferences that were implicit in their stories, which should have been further explored. Preferences related to the theme of ‘Lifestyle’ were expressed clearly in the needs assessments, but in the other four themes ‘Need for activities’, ‘Timing and openness’, ‘Apart or together’, and ‘Meaning of the activity’, we identified latent needs and preferences. The set of indicators with guiding questions is useful for searching actively and systematically for activity needs, including latent needs and their meaning.

### Influence of the study setting: limitations and strengths

The transcripts of the needs assessments used for this study may not represent usual care. Improving tailored care and multi-disciplinary collaboration were important features in the VitaDem project. As a result, the needs assessment got more emphasis than it would in usual care. The dementia case managers were trained to conduct the needs-assessment interviews with a focus on maintaining daily life and ageing in place.

A strength of this study is the inclusion of needs assessment with PWDs. Interviewing PWDs for needs assessment requires a careful approach. Confidence between the PWD and the interviewer and the use of short sentences are important in interviewing PWDs, and questions need to be specific. Questions about needs may be too abstract for them, so prompts and examples were often needed to formulate their experiences, needs, and preferences. Empathy and carefully following their thoughts were essential competencies for the quality of the needs assessments.

### Implications for practice

In a person-centred approach, a professional is sensitive to tracing unmet needs, including activity needs [32]. The professionals’ expertise in client-centred interview skills is a prerequisite for revealing needs and preferences because PWDs and their informal caregivers often express only the needs for which they know a solution exists. They need encouragement to explore latent needs [6, 24, 25]. Our study indicates that explicit training in interview techniques is necessary for professionals in dementia care. However, when unmet needs are made explicit, a higher level of care that adds to health-related quality of life can be achieved [32]. To meet a need adequately, it is also important to explore what that need or wish means to the person. The indicators concerning the ‘Meaning of activities’ may help identify the goals and objectives of a dyad, such as having a fixed routine in the day, just passing time doing something at hand, being physically active, maintaining social contacts, maintaining independency, addressing safety inside and outside the house, or addressing physical limitations that hinder activities. Knowing the meaning of the activities provides clues to finding alternatives if the preferred activity is no longer possible.

Activating interventions aim to increase a dyad’s skills to adapt activities and make changes in the environment or daily routines, and they require readiness [33]. Therefore, the set of indicators include questions about timing and openness for these activating interventions. In an early phase of dementia, PWDs and their informal caregivers are very aware of social and occupational deficits. Nevertheless, assessment is often limited to medical and personal care domains in usual care [4]. Dementia-care specialists plead for integral needs-based approaches for dementia, which are becoming more and more developed and implemented in clinical practice [34–36]. This needs-based approach is also recommended in care pathways for dementia in several West European countries [37, 38]. With regard to activity needs, the potential benefit of activating interventions needs to be taken into account, especially in an early phase of dementia.

### Implications for research

The set of indicators can be a starting point for studying implementation in clinical practice and their utility for professionals. Do they have an added value in exploring activity needs, a dyad’s being ready for change, and estimating the appropriateness of activating interventions? If so, this approach to developing indicators can be applicable for other needs domains and interventions too.

## Conclusions

Needs-based care can contribute to a higher level of care and quality of life for PWDs and their informal caregivers, but it requires good-quality needs assessment. More attention to exploring needs and preferences seems necessary for professionals in dementia care. The indicators can help professionals assess activity needs so that they can better discuss the appropriateness of activating interventions with a dyad.

## Additional file

Additional file 1: Indicators in needs assessment. (DOCX 30 kb)

## Data Availability

See Additional file 1 with a complete overview of the representations of all indicators in the needs assessments.

## Abbreviations

CG: Caregiver (informal)
JG: author Johanna Groenewoud
JL: author Jacomine de Lange
MMSE: Mini Mental State Examination
NL: author Netta Van ‘t Leven
PWD: Person with dementia
PWDs: People with dementia
