# Supplementary material for: Exploring the usefulness of indicators for referring people with dementia and their informal caregivers to activating interventions: a qualitative analysis of needs assessments
Source: BMC Geriatr. 2019 Aug 23;19:230. doi: 10.1186/s12877-019-1221-0 (PMC6706924; doi:10.1186/s12877-019-1221-0)
Supplement: Supplementary file 1 — Indicators in needs assessment. (DOCX 30 kb) [file 12877_2019_1221_MOESM1_ESM.docx]

**Exploring the usefulness of indicators for referring people with dementia and their informal caregivers to activating interventions: a qualitative analysis of needs assessments**

**Additional file 1.** Indicators in needs assessment

| Indicators | | Present in NA (N=20) | Examples and quotes |
| --- | --- | --- | --- |
| *1. Need for activities* | |  |  |
| 1** | The PWD has a need for a meaningful occupational routine. | 14x | People said that they particularly missed some activities and wanted to continue them. Others needed something to do without mentioning anything specific, but wanted a more satisfying way to spend their time.  PWD: *‘I sit here so often with idle hands, and that’s just not my nature.’* |
| 2* | The CG has a need for advice how to cope with the behavior of the PWD. | 9x | Some CGs asked openly for advice. The need for advice was also implicit in statements about expecting too much of the PWDs, asking them to do things they could not do, or blaming them for falling short.  CG: *‘You* [PWD] *tell me that you liked your meal at the day-care centre. So why can’t you remember what it was?’*    CM: *‘She* [PWD] *is inactive and avoids activities. It’s hard for her husband* (CG)*, but he does not know how to activate her.*’ |
| 3* | The CG has a need for support in how to assist or instruct the PWD to perform activities. | 6x | CGs talked about needing advice for practical things like using a mobile phone or remote control, but also more generally, for how to activate the PWD, how to instruct.  CG: *‘When I was ill, I sometimes thought it would be so nice if someone brought me a cup of tea and a biscuit… a few of those little things, you know. But I had to do it all myself.’*  PWD [her husband]: *‘Yes, but I did. It isn’t that I didn’t want to, I just forgot. All those small things I didn’t notice.’*  Other CG: ‘*She* [PWD] *is fanatic about gardening. She can start at 11 a.m., and last week I had to get her from the garden at 8.30 in the evening and I said, ‘Come on, we’re going get ourselves something to eat.’* |
| 4* | The CG has a need for more insight into the capacities of the PWD, what he or she is able to do. | 1x | This indicator was difficult to discern from 1.2.  CM: *‘He* [PWD] *has a room for himself, for his hobbies, which is a mess. His wife wants him to clean it up, but he doesn’t know where to begin.’* |
| *2. Timing and openness for change* | |  |  |
| 1 | The dyad is informed sufficiently about the consequences of dementia for daily life. | 4x | CGs and some PWDs said that they had read about dementia and visited Alzheimer cafés.  PWD: ‘*I feel that I forget things now. I have to watch out not to forget… Yes, I work on it, I write it down.*’ |
| 2 | The CG has an understanding of the consequences of dementia for the daily activities of the PWD. | 2x | Some CGs recognized the consequences because they had other family members with dementia. |
| 3** | The dyad has a pro-active attitude, they want to anticipate themselves on future situations. | 7x | Examples were visiting the Alzheimer cafés or meetings for informal caregivers, searching for information (in books, and on internet), and adapting things in the house to minimalise the risk of falling. The dyad took future changes into account.  CG: *‘We think about what the next step can be. What will be the next thing for her* [PWD] *to hand in? What do we have to know? Where can we get it? Be prepared!*’ |
| 4 | The dyad wants to counteract decline actively as much as possible. | - |  |
| 5** | The dyad is not focused on limitations, but on possibilities. | 9x | Dyads adapted their routines to cope with limitations. Examples were adapting activities, taking a course, using services for groceries, domestic help, and ordering articles online. The dyads also mentioned societal arrangements, e.g. for transport and personal alarms.  CG: *‘I bought an iPad for him. My daughter installed the dementia app and added photos. I have to help him turn it on and find what he wants....’*  PWD adds: *‘You just need to ...eh... put your finger on it, and then the photo of one of my boys, or a photo of her is there* [wife and children].’ |
| 6** | The dyad wants to strongly maintain their current way of living. | 3x | Some dyads did not want to change their routines or could not. They continued their ways as before, despite the consequences of dementia.  CM: ‘*I think she anxiously sticks to this daily program; this is what she can manage.’*  CG: *’I cancelled my domestic help. She never does it the way I want, and then I pay her a pile of money, while I do it myself better and quicker.’* |
| 7 | The PWD still has the capacity to cut out with routines (e.g. use a memory-aid). | - |  |
| 8 | The CG is able to put energy into coping with a new approach, is not overburdened. | 1x | Some CGs said they could take decisions; others were overwhelmed and had little energy for starting an intervention.  CG: *‘I can take decisions firmly and all that.… I hope to continue this for the future. I fear the moment that I have an accident or something. Then it’s game over, for him too* [PWD].*’* |
| *3. Lifestyle* | |  |  |
| 1** | The dyad or one of them (PWD or CG) has or had an active lifestyle. | 8x | The PWDs and CGs talked about their daily activities, now and in the past, painting a picture of an active lifestyle. They mentioned hobbies such as knitting, painting, making music, reading, and physical activity. Others talked about going out *‘getting some fresh air every day’*, meeting their friends, going to the cinema or theatre. Some dyads would baby-sit their grandchildren, some were volunteers in the church or elsewhere. |
| 2** | The PWD and/or CG likes physical activity. | 15x | The PWDs cycled and went for walks. Some mentioned the importance of physical activity for their fitness and for delaying decline; others just liked a walk.  Some dyads had in-house rooms for physical fitness |
| 3* | The PWD and/or CG likes doing sports, in an institutional (group) setting or as a routine (running, bicycling). | 6x | The participants mentioned regularly going to a fitness centre or a cycling club. They also liked regular swimming, golf, and/or yoga |
| 4* | The PWD and/or CG is used to sport. | 5x | Some PWDs and CGs mentioned various sports, such as football, tennis, running, triathlons, handball, and skating.  It was also evident that others were not accustomed to sports.  CG: ‘*I have never been a sportsman or anything like that.’* |
| 5** | The PWD and/or CG likes outings like shopping or making a visit. | 9x | The activities mentioned varied: shopping, going to museums, dancing, going out for dinner, and playing bingo or cards.  PWD: *‘Just sitting around in the house is not my cup of tea.’* |
| *4. Apart or together* | |  |  |
| 1 | The PWD is/is not accustomed to spend time alone. | - |  |
| 2 | The PWD depends a lot/ limited on CG during the day. | 7x | CGs had to manage the PWDs during the day, instruct them, and answer their questions what to do continuously. CGs assisted the PWDs with domestic tasks and, later, personal care as well.  CM: ‘*He* [PWD] *becomes restless if he does not see or hear her.’* |
| 3* | The PWD likes to have enjoyable shared activities with CG. | 3x | Only a few mentioned this:  CM: *‘They are happy with each other. They support each other a lot.’*  CG: *‘Back in those days most of our activities were separate, but now we do a lot more together. I think my wife needs that because of the illness.’* |
| 4* | The CG has a need for enjoyable shared activities with PWD. | 6x | CGs explained what they were accustomed to doing together because of shared interests or practical reasons (domestic tasks). Some missed time to spend together due to appointments.  CG: *‘Cinema, concerts, taking a walk with you* [PWD]… *the prospect of* *some nice things that we can do together. I can look forward to that.’* |
| 5** | The CG has a strong/limited need for his or her own activities. | 8x | CGs mentioned short activities such as leisurely going to a shop or doing a crossword puzzle – and also their hobbies that take time.  CM: *He* [CG] *is a tenor in a choir. He can manage the repetitions in the evening* [leaving his wife alone at home], *but a concert can take a whole day. He foresees that that will be difficult, and he has to make up his mind.’* |
| 6** | The CG has a need for more time for his or her own life. | 10x | The CGs said that they did want to let the PWD be alone for a longer time. They felt rushed too often. Some said they simply needed ‘peace’.  Some CGs cancelled their volunteer work because they could not combine it with caring for their partners.  CG: *‘ I fly to the shop in a hurry, and I fly back again.’* |
| *5. Meaning of activities* | |  |  |
| 1 | The PWD has a strong/limited need for something to do for passing time. | 14x | See part 2.1 |
| 2 | The PWD has a strong/limited need for physical activity. | 15x | See parts 3.2 and 3.3 |
| 3** | The PWD has a strong/limited need for social contacts. | 5x | The PWDs talked about the market, the mall, the sports club, or a club for playing cards because they could meet their friends there and have a chat.  PWD*: I used to have mates whom I met regularly at the garden lots.’* |
| 4** | The PWD has a strong/limited need for self-sufficiency. | 7x | The PWDs said that they wanted to do activities by themselves. This was important for their self-esteem. Some PWDs felt it was import for them to be able to do their share in domestic and household activities.  PWD: *‘I wanted to make the bed, but the sheet… it did not work. It makes me sad. My husband has said it a thousand times: ‘I can do it for you’, but I don’t want that. My answer is: ‘I’ll do it myself.’* |
| 5 | The PWD has a strong/limited need for positive experiences. | - |  |
| 6** | The PWD will benefit from adaptations or assistive devices for physical limitations. | 8x | There were limitations in hand strength, balance, walking, and stairs. Some already had adaptations or assistive devices; others would benefit from them too. |
| 7** | The CG has a need for advice about safety at home. | 2x | Some CGs did not want to leave the PWD alone at home, because of things that had happened, but also because they feared there were other dangerous situations.  CG: *‘Nothing serious has happened – yet. It isn’t that he will burn the house down or anything, but suppose something serious did happen to him.’* |
| 8** | The CG has a need for advice about safety outside. | 2x | CGs were concerned about PWDs not recognising the route and getting lost, and they worried about risky behaviour in traffic.  CG: *‘I definitely do not begrudge her* [PWD] *her cycling, but she is not aware of priority rules, for example.’* |

PWD: person with dementia; CG: caregiver; CM: case manager; NA: needs assessment

** very good recognizable in the majority of clients, and recommended by expert panel (25)

* fairly good recognizable in the majority of clients, and recommended by expert panel (25)
